# Combined use of drug-eluting stent and drug-coated balloon for tandem lesion with spontaneously recanalized coronary thrombus: insights from optical coherence tomography

**DOI:** 10.1093/ehjcr/ytag194

**Published:** 2026-03-12

**Authors:** Satoshi Terashima, Takashi Kataoka, Ken Harada

**Affiliations:** Department of Cardiology, Chubu Rosai Hospital, 10-6 1-chome Komei Minato-ku, Nagoya, Aichi 455-8530, Japan; Department of Cardiology, Chubu Rosai Hospital, 10-6 1-chome Komei Minato-ku, Nagoya, Aichi 455-8530, Japan; Department of Cardiology, Chubu Rosai Hospital, 10-6 1-chome Komei Minato-ku, Nagoya, Aichi 455-8530, Japan

## Case description

**Figure 1:**
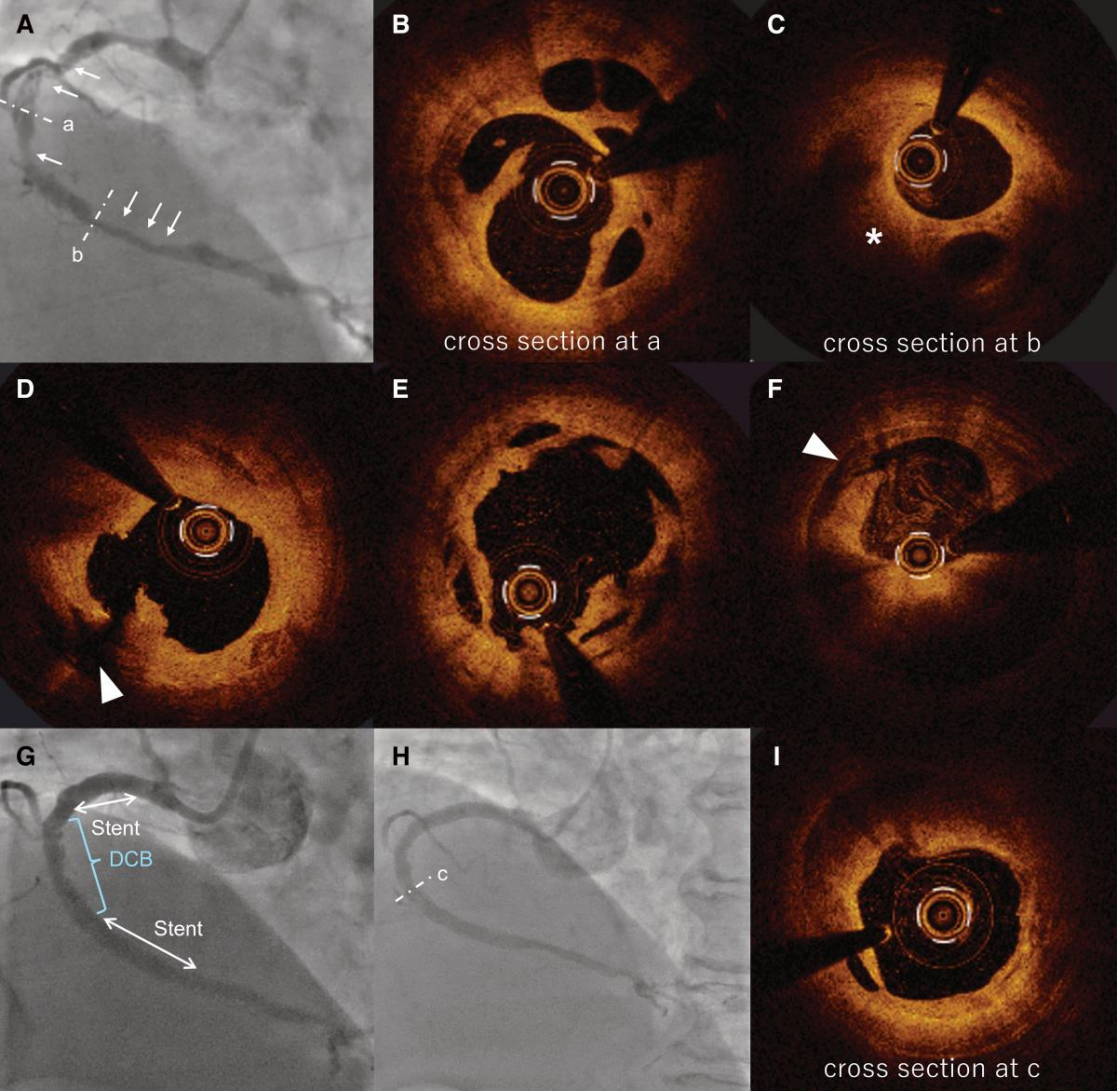
(*A*) Coronary angiography indicating tandem lesion (arrows) with a braid-like appearance in both the proximal and distal of RCA. (*B*) OCT imaging from cross section at (*A*) showing a lotus root-like appearance. (*C*) OCT imaging from cross section at (*B*) showing a lotus root-like appearance and mural thrombi (asterisk). (*D*) Cross-sectional OCT image of the proximal RCA after lesion preparation, demonstrating a major dissection extending into media (arrowhead). (*E*) Cross-sectional OCT image at (*A*) after preparation, showing well-controlled dilatation and an adequately enlarged lumen. (*F*) Cross-sectional OCT image at (*B*) after preparation, demonstrating a major dissection extending into the media (arrowhead) and residual plaque volume. (*G*) Final angiography of PCI showing lesions treated with DES and DCB. (*H*) Coronary angiography at the one-year follow-up. (*I*) OCT imaging from the cross-section at (*C*), which had been treated with DCB. DCB, drug-coated balloon; DES, drug-eluting stent; MLA, minimum lumen area; OCT, optical coherence tomography; PCI, percutaneous coronary intervention; RCA, right coronary artery.

A 70-year-old woman with type 2 diabetes mellitus presented with progressive chest pain on exertion following an episode of sudden chest pain at rest two weeks earlier. Coronary angiography revealed a severe stenosis with a braid-like appearance extending from proximal to distal segments of the right coronary artery (RCA) (*Figure 1A*, arrows). Angiography suggested a spontaneously recanalized coronary thrombus (SRCT), which previous reports indicate is often physiologically significant.^1^

Optical coherence tomography (OCT)-guided percutaneous coronary intervention (PCI), which is associated with favourable outcomes,^2^ was performed. OCT imaging revealed a tandem lesion with lotus root-like appearances and mural thrombi (*Figure 1C*, asterisk) extending over 100 mm (*Figure 1B* and *C*). Although optimal PCI strategy for SRCT is not well established, drug-eluting stents (DES) implantation has shown promising results, and drug-coated balloons (DCB) may offer an alternative.^3^ Following adequate lesion preparation with a cutting balloon, angiography showed residual stenosis greater than 50% in proximal and distal segments while mid segment had comparatively less stenosis. Additionally, OCT revealed that the proximal and distal lesions had developed major dissections extending to the media (*Figure 1D* and *F*, arrowheads), whereas the mid lesion demonstrated a well-controlled dissection with relatively less plaque volume (*Figure 1E*). To minimize risks associated with full coverage of RCA using multiple overlapping DES, a hybrid approach using DES and DCB was adopted (*Figure 1G*).

One year after treatment, she developed exertional dyspnoea with progression in the left anterior descending artery. OCT-guided PCI was performed for the *de novo* lesion, and RCA was examined. Angiography of RCA demonstrated sustained patency of the treated lesion (*Figure 1H*). OCT imaging at the DCB-treated site showed favourable expansion at minimum lumen area (*Figure 1I*). This case highlights the successful use of combined DES and DCB therapy for extensive SRCT, suggesting its potential as a viable treatment strategy.

## Supplementary Material

ytag194_Supplementary_Data

## Data Availability

The underlying data are available in the article or the Supplementary material online, material.

## Notes

**Consent:** The authors confirm written informed consent was obtained from the patient for the publication of this case report including images in accordance with the COPE guidance.
